# Nicotinamide Adenine Dinucleotide Protects against Spinal Cord Ischemia Reperfusion Injury-Induced Apoptosis by Blocking Autophagy

**DOI:** 10.1155/2017/7063874

**Published:** 2017-03-07

**Authors:** Lei Xie, Sifei Yu, Zhenfei Wang, Kai Yang, Zhuochao Liu, Changwei Li, Yu Liang

**Affiliations:** ^1^Department of Orthopedics, Ruijin Hospital, Shanghai Jiao Tong University School of Medicine, 197 Ruijin 2nd Road, Shanghai 200025, China; ^2^Shanghai Key Laboratory for Prevention and Treatment of Bone and Joint Diseases with Integrated Chinese-Western Medicine, Shanghai Institute of Traumatology and Orthopedics, Ruijin Hospital, Shanghai Jiao Tong University School of Medicine, 197 Ruijin 2nd Road, Shanghai 200025, China

## Abstract

The role of autophagy, neuroprotective mechanisms of nicotinamide adenine dinucleotide (NAD^+^), and their relationship in spinal cord ischemic reperfusion injury (SCIR) was assessed. Forty-eight Sprague-Dawley rats were divided into four groups: sham, ischemia reperfusion (I/R), 10 mg/kg NAD^+^, and 75 mg/kg NAD^+^. Western blotting, immunofluorescence, and immunohistochemistry were used to assess autophagy and apoptosis. Basso, Beattie, and Bresnahan (BBB) scores were used to assess neurological function. Expression levels of Beclin-1, Atg12-Atg5, LC3B-II, cleaved caspase 3, and Bax were upregulated in the I/R group and downregulated in the 75 mg/kg NAD^+^ group; p-mTOR, p-AKT, p62, and Bcl-2 were downregulated in the I/R group and upregulated in the 75 mg/kg NAD^+^ group. Numbers of LC3B-positive, caspase 3-positive, Bax-positive, and TUNEL-positive cells were significantly increased in the I/R group and decreased in the 75 mg/kg NAD^+^ group. The mean integrated option density of Bax increased and that of Nissl decreased in the I/R group, and it decreased and increased, respectively, in the 75 mg/kg NAD^+^ group. BBB scores significantly increased in the 75 mg/kg NAD^+^ group relative to the I/R group. No difference was observed between I/R and 10 mg/kg NAD^+^ groups for these indicators. Therefore, excessive and sustained autophagy aggravates SCIR; administration of NAD^+^ alleviates injury.

## 1. Introduction

Spinal cord ischemia reperfusion (I/R) injury (SCIR) is a disastrous complication in many pathophysiological situations, which may result in devastating paraplegia and paraparesis [1]. SCIR has been reported to occur in 3%–18% of patients undergoing descending thoracic and thoracoabdominal aneurysms surgery and endovascular aortic repair surgery [2]. SCIR should be regarded not only as a medical problem, but also as a socioeconomic burden. Considerable therapeutic interventions have been attempted to mitigate this problem, including cerebrospinal fluid drainage, reattachment of segmental arteries, intercostal vessel reimplantation [3, 4], and administering pharmaceuticals (steroids, oxygen-derived free radical scavengers, and vasodilators) and drugs [5, 6]. However, the results were not satisfactory [7].

Autophagy plays an important role for survival when cells encounter metabolic stress and for metabolic processes maintaining cytoplasmic compositions via the autophagosomal-lysosomal pathway. Autophagy is an intracellular catabolic mechanism which can promote cell survival through degrading and recycling damaged organelles and unwanted proteins but also induce cell death in some pathological conditions [8]. Currently, several studies have been conducted to explore the functional role of autophagy in SCIR. However, the results of these studies are inconsistent. Some studies reported that activation and upregulation of autophagy reduce nerve damage after I/R [9, 10], whereas other studies found that autophagy contributed to neuronal death and inhibition of autophagy seemed to provide protective effects [11, 12]. In general, the precise role of autophagy remains controversial in SCIR and requires further investigation.

Nicotinamide adenine dinucleotide (NAD^+^) is a vital cofactor for metabolizing energy and a substrate for various enzymes [13]. Mounting evidence suggests that NAD^+^ plays an essential role in calcium homeostasis, mitochondrial, and immunological functions [14, 15]. Previous studies showed that cerebral I/R results in reduction of NAD^+^ levels [16, 17]. Repletion of NAD^+^ could alleviate I/R injury and prevent astrocyte death in the brain [18]. In a previous study, we have shown that NAD^+^ protects against SCIR injury via reducing oxidative stress-induced neuronal apoptosis [19]. However, whether NAD^+^ can alleviate SCIR injury by restraining autophagic apoptosis remains to be elucidated.

The primary goal of our study was to investigate the role of autophagy and its potential signaling pathway in SCIR. Furthermore, we aimed to examine the protective effect and the internal mechanism of NAD^+^ involved in SCIR, as well as to explore the underlying relationship between autophagy and NAD^+^.

## 2. Materials and Methods

### 2.1. Animals

Forty-eight male Sprague-Dawley rats (180–250 g body weight), obtained from the Shanghai Laboratory Animal Corporation (Shanghai, China), were used in this study. The rats were acclimatized to laboratory conditions (25°C, 12 h/12 h light/dark, 50% humidity, and ad libitum access to food and water) for one week prior to experiments. All procedures involving animals were reviewed and approved by the Institutional Animal Care and Use Committee of Ruijin Hospital, Shanghai Jiao Tong University, Shanghai, China [IACUC protocol number: SYXK (Shanghai) 2011-0113].

### 2.2. Surgical Procedure of Spinal Cord Ischemic Reperfusion Injury

SCIR was modeled using a modified method from Zhang et al. [20]. Briefly, all rats were neurologically intact before the experiment and anesthetized with 2.5% sodium pentobarbital (60 mg/kg) administered intraperitoneally. After opening the retroperitoneum through the midline incision, the abdominal aorta was blocked above the right renal artery near the heart using a 50 g aneurysm clip for 60 min. The clips were removed just before closure. Sham-operated rats underwent the same procedure, but no occlusion of the aorta was performed. All operated rats were placed in a box at 28°C until anesthetic recovery and subsequently placed in separated cages with ad libitum access to food and water.

### 2.3. Experimental Protocol

A total of 48 rats were randomly divided into four groups. The sham group (*n* = 12) underwent the surgical procedure without aortic clipping. The I/R group (*n* = 12) received abdominal aortic exposure and cross-clamping for 60 min followed by intraperitoneal injection of equivalent volume of 0.9% saline solution immediately after reperfusion. Rats in the 10 mg/kg NAD^+^ group (*n* = 12) and 75 mg/kg NAD^+^ group (*n* = 12) underwent the same surgical procedure as those in the I/R group but were treated with different doses of NAD^+^ immediately after I/R injury.

### 2.4. NAD^+^ Preparation and Treatment

NAD^+^ (Roche, Mannheim, Germany) was dissolved in 0.9% saline solution and injected intraperitoneally. To investigate whether NAD^+^ has protective effects in SCIR, rats were treated with 10 mg/kg and 75 mg/kg NAD^+^ intraperitoneally. The control group was treated with equivalent volume of 0.9% saline solution. Dosage of NAD^+^ was decided based on previous studies with minor modification [19, 21].

### 2.5. Tissue Preparation

A total of 24 rats (randomly 6 rats from each group) were euthanized 24 h after reperfusion by an intraperitoneal injection of overdosed (100 mg/kg) sodium pentobarbital. The rats were transcardially perfused with 0.9% saline, followed by 4% paraformaldehyde in 0.1 M PBS, pH 7.4. Spinal cord segments (L1-2) were collected, post-fixed in the same fixative overnight at 4°C, and divided into two parts. One part was embedded in paraffin and the other was embedded in optimal cutting temperature (OCT) compound (Sakura, Torrance, USA). Serial, 6 *μ*m, transverse sections were mounted on slides. Paraffin sections were used for histology and frozen sections for immunofluorescence and terminal deoxynucleotidyl transferase-mediated dUTP nick end labeling (TUNEL) as described below. The L1-2 segments of spinal cords of the remaining 24 rats were rapidly collected and deep-frozen at −80°C in preparation for western blot analysis.

### 2.6. Assessment of Neurological Function

Locomotor function of rats was recorded at different time points (1, 6, 12, and 24 h) after reperfusion using the Basso, Beattie, and Bresnahan (BBB) open-field locomotor scale [22] ranging from 0 (complete paralysis) to 21 (normal locomotion). Two blind observers scored each animal, respectively, and any discrepancies between the two scores were resolved by discussion.

### 2.7. Nissl Staining

All paraffin sections were deparaffinized and rehydrated. The sections were washed in distilled water three times, then stained with 0.25% toluidine blue at 50°C for 3 h, and bleached using 95% ethanol. Subsequently, sections underwent 100% ethanol dehydration, xylene transparency, and neutral gum mounting. In each experiment, all sections were stained at the same time.

### 2.8. Immunohistochemical Staining of Bax

Paraffin sections were deparaffinized and rehydrated. Antigen retrieval was performed according to the manufacturer's instructions for the Citrate Antigen Retrieval Solution (Beyotime, China). Sections were incubated in hydrogen peroxide to quench any endogenous peroxidases and then blocked with 5% bovine serum albumin (Sigma) for 30 min at room temperature. Sections were incubated in primary rabbit anti-Bax antibodies (1 : 100; Abcam) diluted in PBS overnight at 4°C. The Vectastain Elite ABC Kit (Vector Laboratories, USA) was used according to the manufacturer's instructions. Positive staining was visualized with DAB (ImmPACT DAB, Vector Laboratories, USA). Sections were counterstained with hematoxylin for 10 s and dipped in acid alcohol as needed before being dehydrated and mounted on coverslips.

### 2.9. Counting and Calculation of Mean Integrated Option Density of Nissl and Bax

Digital photographs of all sections were taken using a ZEISS Axioskop microscope. The images were analyzed using Image-Pro Plus 6.0 software (Media Cybernetics, USA), and sum of area and integrated option density (IOD) were measured. The mean integrated option density was calculated by dividing the IOD sum by the area sum. The above information was collected in three sections for each rat by three blind observers.

### 2.10. Immunofluorescence Staining of Bax, Caspase 3, and LC3

Frozen sections were washed with PBS for 10 min and then with PBS containing 0.1% Tween (PBST) for 10 min and then blocked with 5% bovine serum albumin (Sigma) for 30 min at room temperature. Sections were incubated in permeabilization solution (1% Triton X-100) for 15 min at room temperature and then incubated in primary rabbit anti-Bax antibodies (1 : 100; Santa Cruz Biotechnology), primary rabbit anti-caspase 3 antibodies (1 : 100; Santa Cruz Biotechnology), or primary rabbit anti-LC3B antibodies (1 : 200; Cell Signaling Technology) diluted in PBS overnight at 4°C. After rinsing with PBST, sections were incubated with goat anti-rabbit IgG Alexa Fluor 488 secondary antibody (1 : 500; Molecular Probes) for 2 h at room temperature. The sections were mounted with ProLong® Gold antifade reagent with DAPI to label the nuclei (Molecular Probes).

### 2.11. Terminal Deoxynucleotidyl Transferase-Mediated dUTP Nick End Labeling (TUNEL) Assay

In order to identify DNA fragmentation, TUNEL was performed. Apoptotic cells in the frozen spinal cord sections were stained using the in situ Cell Death Detection kit (TMR red; Roche Diagnostics GmbH), according to the manufacturer's instructions. Briefly, sections were washed in PBS and incubated in permeabilization solution for 15 min at room temperature and then in the TUNEL solution containing TMR-dUTP for 1 h at 37°C. After labeling, cell nuclei were labeled with ProLong Gold antifade reagent with DAPI (Molecular Probes). After the immunofluorescence staining protocol described above, all sections were scanned using a confocal microscope (LSM 710, ZEISS, Germany). To quantify Bax, caspase 3, LC3 expression levels, and TUNEL in the spinal cord, labeled cells and mean fluorescence intensity were recorded in each spinal cord transverse section. The average of the numbers in the three sections was compared between groups. The above analysis was performed by three blinded investigators.

### 2.12. Western Blot Analysis

L1-2 segments of spinal cords were homogenized in Radio-Immunoprecipitation Assay (RIPA) buffer (Beyotime, China) with phenylmethanesulfonyl fluoride (PMSF) protease and Phosphatase Inhibitor Cocktail (CWBIO, China). Homogenates were clarified using centrifugation at 12000 ×g for 15 min at 4°C. The concentration of protein samples was determined using the BCA protein assay kit (Beyotime, China). Aliquots of protein (50 *μ*g/lane) were fractionated using 10% sodium dodecyl sulfate polyacrylamide gel electrophoresis (SDS-PAGE). After electrophoresis, the proteins on the gel were transferred to polyvinylidene difluoride membranes (0.45 *μ*m, Millipore, USA). The membranes were blocked in Tris-buffered saline/Tween (20 mmol/L Tris, pH 7.5, 0.5 mol/L NaCl, and 0.1% Tween 20) containing 5% nonfat dry milk for 1 h at room temperature and subsequently incubated with primary antibodies overnight at 4°C. Membranes were incubated with secondary antibody for 90 min at room temperature. Chemiluminescence results were recorded using an imaging system (Imagequant LAS4000mini, General Electric, USA). Signal intensities were quantified using Image-Pro Plus software. The antibodies used were as follows: rabbit anti-mTOR (1 : 1000; Cell Signaling Technology), rabbit anti-p-mTOR (1 : 1000; Cell Signaling Technology), rabbit anti-AKT (1 : 1000; Cell Signaling Technology), rabbit anti-p-AKT (1 : 1000; Cell Signaling Technology), mouse anti-SQSTM1/p62 (1 : 1000; Abcam), rabbit anti-Atg12 (1 : 1000; Cell Signaling Technology), rabbit anti-cleaved caspase 3 (1 : 1000; Cell Signaling Technology), rabbit anti-Bcl-2 (1 : 1000; Cell Signaling Technology), rabbit anti-Bax (1 : 1000; Cell Signaling Technology), rabbit anti-LC3B (1 : 1000; Cell Signaling Technology), mouse anti-*β*-actin (1 : 1000; Cell Signaling Technology), and horseradish peroxidase- (HRP-) conjugated secondary antibodies (1 : 5000; Jackson).

### 2.13. Statistical Analysis

All data were expressed as mean ± standard deviation (mean ± SD) and analyzed by Statistical Package for Social Sciences software (version 19.0). Groups were analyzed using the one-way analysis of variance (ANOVA), followed by Newman-Keuls post hoc analysis. Threshold for statistical significance was set at *P* < 0.05.

## 3. Results

### 3.1. NAD^+^ Inhibits Autophagy Activation after I/R Injury

**Figure 1:**
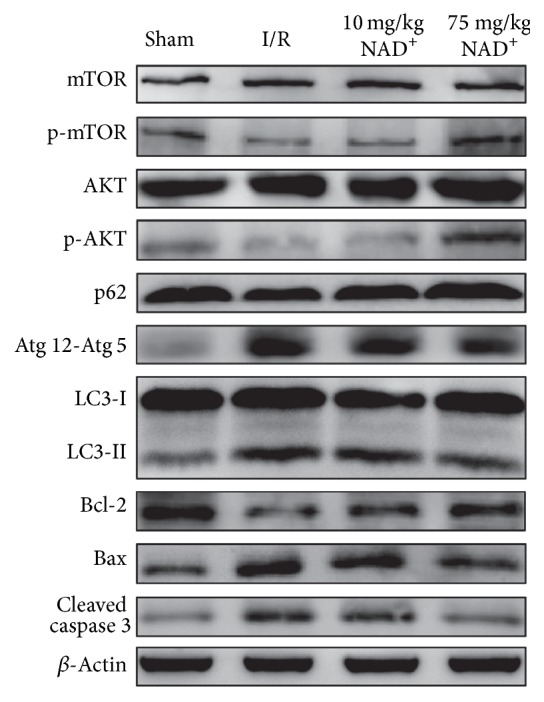
Changes in protein expression after spinal cord ischemia reperfusion (I/R) injury and treatment with different dose of nicotinamide adenine dinucleotide (NAD^+^) (*n* = 6 per group).

**Figure 2:**
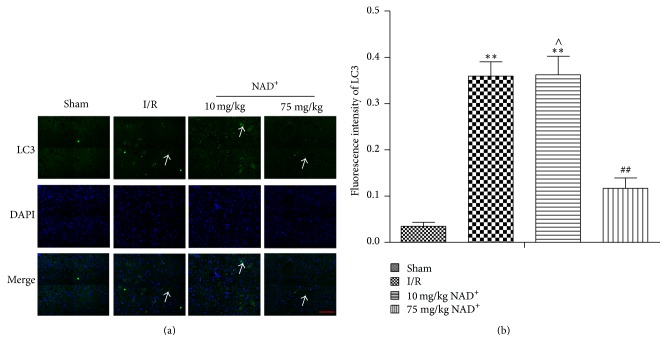
Immunofluorescence staining of LC3B in sham, ischemia reperfusion (I/R), 10 mg/kg nicotinamide adenine dinucleotide (NAD^+^), and 75 mg/kg NAD^+^ group. (a) LC3B-positive neurons (arrow) were observed in different groups (400x, scale bar = 50 *μ*m). (b) Analysis of fluorescence intensity of LC3B-positive neurons in each group. Compared with the sham group, the fluorescence intensity of LC3B-positive neurons was significantly increased in the I/R group. Compared with the I/R group, the fluorescence intensity of LC3B-positive neurons was significantly decreased in the 75 mg/kg NAD^+^ group. There was no significant difference in 10 mg/kg NAD^+^ group. Values are means ± SD (*n* = 6 per group). ^*∗∗*^*P* < 0.01 versus sham; ^##^*P* < 0.01 versus I/R; ^∧^*P* > 0.05 versus I/R.

Our previous study [19] revealed that NAD^+^ with the concentration higher than 50 mg/kg significantly alleviated spinal cord ischemia-reperfusion injury via reducing oxidative stress-induced neuronal apoptosis; however, 10 mg/kg NAD^+^ showed no obvious therapeutic effect on this damage. So, we chose 75 mg/kg NAD^+^ as an effective therapeutic dosage and chose 10 mg/kg NAD^+^ as an invalid ineffective concentration in this study. To detect whether NAD^+^ exerted an effect on autophagy activation in SCIR injury, it is essential to measure the level of protein LC3, p62, and Atg12-Atg5 proteins. Our result shows that expression levels of LC3-II in spinal cords of the I/R group were significantly larger at 24 h than in sham-operated rats. Treatment with 75 mg/kg NAD^+^ could decrease the LC3-II protein expression at 24 h. However, no significant difference was observed between the 10 mg/kg NAD^+^ group and the I/R group (Figure 1). Atg12-Atg5 was upregulated in the I/R and 10 mg/kg NAD^+^ group and downregulated in the 75 mg/kg NAD^+^ group (Figure 1). However, LC3-II gave similar results. Conversely, the expression of p62 decreased in the I/R and 10 mg/kg NAD^+^ group. However, no significant decrease in expression was observed in the 75 mg/kg NAD^+^ group (Figure 1). The above changes in protein expression could indicate an activation of autophagy in SCIR. To further explore autophagy activation in neuronal cells, immunofluorescence staining of LC3 was performed (Figure 2(a)). The results showed that mean fluorescence intensity of LC3 increased significantly in neuronal cells of the I/R group (*P* < 0.01, versus sham), indicating autophagy activation in neurons. Compared with the I/R group, the mean fluorescence intensity of LC3 decreased in the 75 mg/kg NAD^+^ group (*P* < 0.01). No significant difference was detected in the 10 mg/kg NAD^+^ group compared with I/R group (*P* > 0.05; Figure 2(b)). These results confirmed that autophagy was activated in neuronal cells after SCIR. Administration of NAD^+^ could inhibit autophagy to a certain extent.

### 3.2. NAD^+^ Ameliorates Deteriorated Neurological Functions after Autophagy Activation after I/R Injury

**Figure 3:**
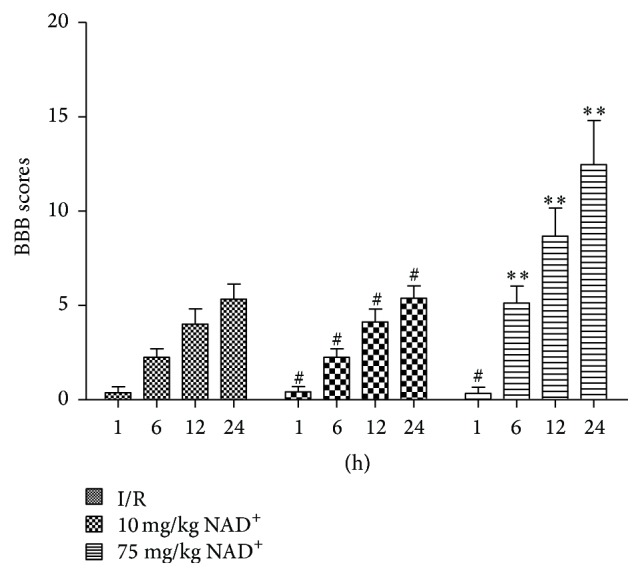
Basso, Beattie, and Bresnahan (BBB) scores of rats in ischemia reperfusion (I/R), 10 mg/kg nicotinamide adenine dinucleotide (NAD^+^), and 75 mg/kg NAD^+^ group at each time point. BBB scores were measured from 1 h to 24 h after I/R injury. Compared with the I/R group, BBB scores were consistently higher in 75 mg/kg NAD^+^-treated rats from 6 to 24 h after I/R injury, whereas there were no significant differences in the 10 mg/kg NAD^+^ group from 1 h to 24 h after injury. Values are means ± SD (*n* = 12 per group). ^*∗∗*^*P* < 0.01 versus I/R; ^#^*P* > 0.05 versus I/R.

**Figure 4:**
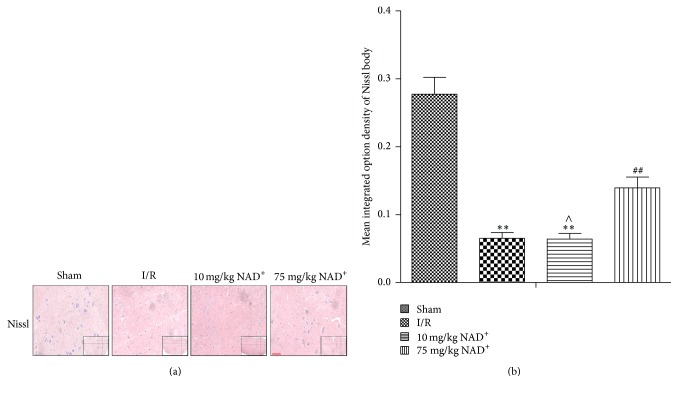
Nissl staining in sham, ischemia reperfusion (I/R), 10 mg/kg NAD^+^, and 75 mg/kg nicotinamide adenine dinucleotide (NAD^+^) group. (a) Nissl bodies were observed in different groups (100x, scale bar = 100 *μ*m). (b) Analysis of the mean integrated option density (IOD) of Nissl bodies in each group. Compared with the sham group, the mean IOD of Nissl bodies was significantly decreased in the I/R group. Compared with the I/R group, the mean IOD of Nissl bodies was significantly increased in the 75 mg/kg NAD^+^ group. No significant differences were observed in the 10 mg/kg NAD^+^ group. Values are means ± SD (*n* = 6 per group). ^*∗∗*^*P* < 0.01 versus sham; ^##^*P* < 0.01 versus I/R; ^∧^*P* > 0.05 versus I/R.

Motor functions of rats were evaluated using BBB scores.  Figure 3 showed the trends in the different groups. All rats experienced severe paraplegia in the first few hours after reperfusion, except rats from the sham group. The 75 mg/kg NAD^+^ group showed faster and better recovery of motor functions than the I/R group (*P* < 0.01); however, I/R rats treated with 10 mg/kg NAD^+^ showed no significant improvement in motor functions compared with the I/R group (*P* > 0.05). These results were in accordance with results from Nissl staining (Figure 4(a)). The mean IOD of Nissl was significantly smaller in the I/R and 10 mg/kg NAD^+^ group (*P* < 0.01, versus sham) and larger in the 75 mg/kg NAD^+^ group compared with I/R group (*P* < 0.01; Figure 4(b)). These results indicate that neurological function deteriorated after I/R injury and was gradually restored with the administration of NAD^+^.

### 3.3. Autophagy Can Aggravate and NAD^+^ Can Alleviate Cell Apoptosis in I/R Injury

**Figure 5:**
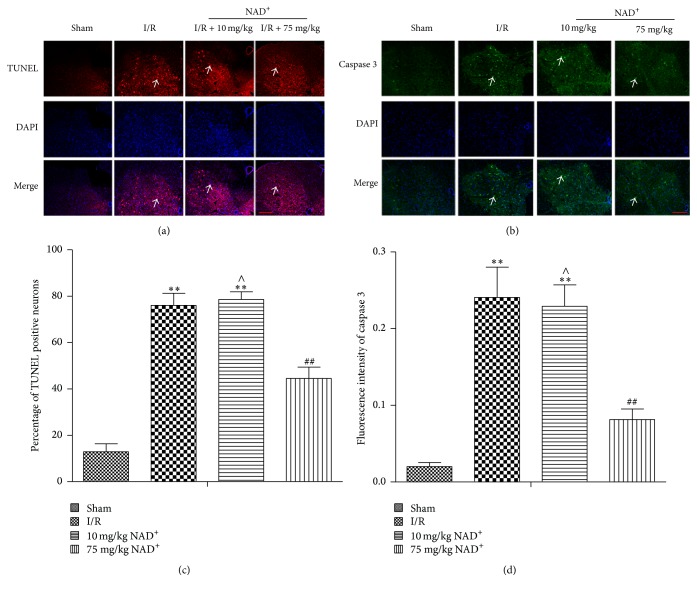
Immunofluorescence staining of terminal deoxynucleotidyl transferase-mediated dUTP nick end labeling (TUNEL) and caspase 3 in sham, ischemia reperfusion (I/R), 10 mg/kg nicotinamide adenine dinucleotide (NAD^+^), and 75 mg/kg NAD^+^ group. (a) TUNEL-positive neurons (arrow) were observed in different groups (200x, scale bar = 100 *μ*m). (b) Caspase 3-positive neurons (arrow) were observed in different groups (200x, scale bar = 100 *μ*m). (c, d) Analysis of the percentage of TUNEL-positive neurons and fluorescence intensity of caspase 3-positive neurons in each group, respectively. Compared with the sham group, the percentage of TUNEL-positive neurons and fluorescence intensity of caspase 3-positive neurons were significantly increased in the I/R group. Compared with the I/R group, the percentage of TUNEL-positive neurons and fluorescence intensity of caspase 3-positive neurons significantly were decreased in the 75 mg/kg NAD^+^ group. No significant differences were observed in the 10 mg/kg NAD^+^ group. Values are means ± SD (*n* = 6 per group). ^*∗∗*^*P* < 0.01 versus sham; ^##^*P* < 0.01 versus I/R; ^∧^*P* > 0.05 versus I/R.

To investigate the interaction between autophagy and apoptosis in ischemic reperfusion injury, we examined the protein expression levels of cleaved caspase 3 and immunofluorescence signals of TUNEL and caspase 3. Our results indicate that expression of cleaved caspase 3 was distinctly lower in the 75 mg/kg NAD^+^ group than the I/R and 10 mg/kg NAD^+^ group (Figure 1). Immunofluorescence staining gave similar results (Figures 5(a) and 5(b)). Greater number of TUNEL-positive neurons and higher mean fluorescence intensity of caspase 3 were detected in the I/R and 10 mg/kg NAD^+^ group (*P* < 0.01 versus sham). In contrast, both parameters were dramatically lower in the 75 mg/kg NAD^+^ group (*P* < 0.01; Figures 5(c) and 5(d)). Therefore, we concluded that autophagy aggravated neuronal apoptosis in I/R injury and NAD^+^ decreased neuronal apoptosis.

### 3.4. The Pathway of Autophagy Activation and Neuroprotective Mechanisms of NAD^+^

**Figure 6:**
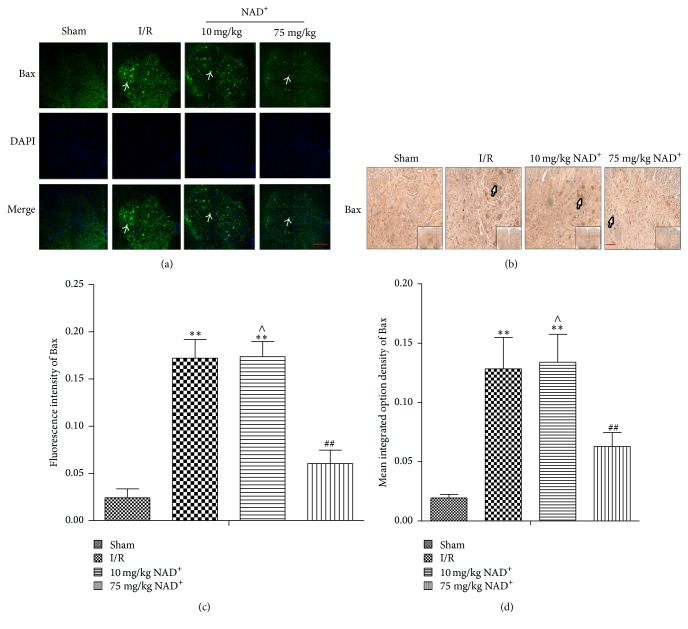
Immunofluorescence and immunohistochemical staining of Bax in sham, ischemia reperfusion (I/R), 10 mg/kg nicotinamide adenine dinucleotide (NAD^+^), and 75 mg/kg NAD^+^ group. (a) Bax-positive neurons (arrow) were observed in different groups (200x, scale bar = 100 *μ*m). (b) Bax-positive neurons (arrow) were observed in different groups (100x, scale bar = 100 *μ*m). (c, d) Analysis of fluorescence intensity of Bax-positive neurons and the mean integrated option density (IOD) of Bax in each group, respectively. Compared with the sham group, the fluorescence intensity of Bax-positive neurons and the mean IOD of Bax were significantly increased in the I/R group. Compared with the I/R group, the fluorescence intensity of Bax-positive neurons and the mean (IOD) of Bax-positive neurons were significantly decreased in the 75 mg/kg NAD^+^ group and were not significantly different in the 10 mg/kg NAD^+^ group. Values are means ± SD (*n* = 6 per group). ^*∗∗*^*P* < 0.01 versus sham; ^##^*P* < 0.01 versus I/R; ^∧^*P* > 0.05 versus I/R.

The PI3K/AKT/mTOR pathway is an intracellular signaling pathway important in regulating the cell cycle. A recent study has shown that AKT/mTOR signaling pathway is essential in the activation of autophagy [8]. To explore the role of the AKT/mTOR pathway, western blot analysis was performed. Our results indicate that expression of p-AKT and p-mTOR was significantly lower in I/R and 10 mg/kg NAD^+^ group, but markedly higher in the 75 mg/kg NAD^+^ group. The Bcl-2 family of apoptosis-related genes has been considered to play a central role in regulating apoptotic signaling cascade [23]. Bcl-2 exerts a survival function in response to a wide range of apoptotic stimuli through inhibition of mitochondrial cytochrome c release. Moreover, Bax is a key component for cellular induced apoptosis through mitochondrial stress. Therefore, we further evaluated whether the expression of these proteins was correlated with apoptosis. We found the increased Bax and decreased Bcl-2 in I/R and I/R + 10 mg/kg NAD^+^ group, and the processes were significantly restrained after 75 mg/kg NAD^+^ administration (Figure 1). Immunofluorescence and immunohistochemistry analysis of Bax further confirmed these results (Figure 6). Taken together, our data suggests that autophagy is partially regulated through the PI3K/AKT/mTOR pathway and apoptosis is partially regulated through the Bcl-2/Bax pathway. Furthermore, the protective effect of NAD^+^ may occur in part through these pathways.

## 4. Discussion

SCIR involves a series of complex metabolic derangements including molecular and cellular events, such as oxidative stress, inflammatory response, apoptosis, and autophagy [24]. In addition to primary ischemic injury, neuronal death induced by damage secondary to reperfusion is the major therapeutic conundrum in SCIR [25]. A growing body of experimental studies described the pathological derangement and molecular and cellular events in ischemic reperfusion injury and examined the efficacy of novel strategies [26–28]. A role of autophagy has been implicated not only in central nervous system diseases, including traumatic brain injury, cerebral ischemia, and neurodegeneration [28, 29], but also in other pathological conditions, such as cancer, myocardial infarction, and infections [26, 30]. However, the exact role of autophagy in these processes remains to be elucidated. Recently, an increasing number of studies have investigated the role of autophagy in I/R injury. Whether autophagy is neuroprotective in spinal cord injury has not yet been clarified. Therefore, we aimed to explore the types of neuronal death and potential signaling pathways involved in SCIR. An understanding of these mechanisms is critical for reliable protective therapeutic strategies.

Mounting evidence suggests that NAD^+^ plays an important role in mitochondrial function, calcium homeostasis, and immunological functions [14, 15]. NAD^+^ is a key cofactor for metabolizing energy and various enzymes [13]. NAD^+^ repletion has been suggested to decrease astrocyte death [18]. However, studies regarding the effect of NAD^+^ on SCIR and the role of NAD^+^ involved in autophagy are scarce. In this study, for the first time, we investigate the role of autophagy in SCIR and assess the effect of NAD^+^ administration after SCIR. We found that expression of LC3-II was significantly increased in the I/R group and decreased in the 75 mg/kg NAD^+^ group. Moreover, the level of p62 expression was decreased in I/R group, whereas it remained unchanged in the 75 mg/kg NAD^+^ group. Moreover, the mean fluorescence intensity of LC3 was significantly increased in neurons of the I/R group and decreased in the 75 mg/kg NAD^+^ group. These findings suggested that autophagy occurred after SCIR and that a high enough level of NAD^+^ could inhibit it.

In this study, we focused on exploring the relationship between apoptosis and autophagy after SCIR. Our results show that cleaved caspase 3 was obviously increased in the I/R group and reduced in the 75 mg/kg NAD^+^ group relative to the I/R group. The TUNEL and caspase 3 positive cells were neurons. Furthermore, the number of TUNEL-positive cells and mean fluorescence intensity of caspase 3 also increased in the I/R group and decreased in 75 mg/kg NAD^+^ group. In conjunction with previous findings, we propose that activation of autophagy might promote neuronal apoptosis, whereas NAD^+^ administration reduced neuronal loss by blocking autophagy. These results seem to contradict the results of some previous studies, which concluded that autophagy activation could prevent neuronal loss and had antiapoptotic effects in spinal cord injury [9, 10]. There are several possible explanations for the discrepancy: First, different experimental models were used to assess the function of autophagy and its relationship with apoptosis. Second, the degree of autophagy activation differs between the different studies, suggesting that autophagy may play a dual role: low levels of autophagy activation promote cell survival, while overactivation of autophagy leads to cell death. Surgical method, ischemic time, and reperfusion time are also potential confounding factors. This interpretation also explains the complex role of autophagy in SCIR. Further well-designed studies are warranted to clarify this issue.

Next, we focused on the signaling pathway activating autophagy and the protective mechanism of NAD^+^. The formation of the autophagosome, elongation, maturation, and fusion are the major steps of autophagy. Various proteins are involved in this process; however only a small subset has been identified until now [31]. The PI3K/AKT/mTOR pathway is an intracellular signaling pathway important in cell cycle regulation. PI3K/AKT/mTOR pathway is the most classical activation pathway of autophagy. Akt activates the downstream mTOR signaling pathway. mTOR is inactivated by nutrient deprivation and then regulates downstream proteins to facilitate nucleation, the first step of autophagic vesicle formation [8]. The molecular mechanism of mTOR regulation in SCIR is still controversial. In this study, we found that phosphorylation of AKT and mTOR was inhibited in I/R group and activated in the 75 mg/kg NAD^+^ group. We also analyzed expression levels of Atg12-Atg5, p62, and LC3B. Our results supported the above hypothesis that PI3K/AKT/mTOR is included in the activation pathway of autophagy in SCIR. Furthermore, we investigated Beclin-1 expression, because previous studies have indicated that Beclin-1-independent autophagy was alternative pathway [8, 32, 33]. Our results confirmed these observations. Taken together, both PI3K/AKT/mTOR and Beclin-1-independent pathway regulated autophagy and higher doses of NAD^+^ could block these two pathways.

Previous studies have shown that Bax can induce apoptosis, whereas Bcl-2 exerts an antiapoptotic effect [23]. In the present study, we assessed the expression of Bcl-2 and Bax and immunofluorescence/immunohistochemistry signals of Bax. In the I/R group, expression of Bcl-2 decreased, whereas expression of Bax, mean fluorescence intensity, and mean IOD of Bax increased. Opposite effects were observed in 75 mg/kg NAD^+^ group compared with the I/R group. These findings indicate that the Bcl-2/Bax pathway is related to neuronal apoptosis in SCIR and NAD^+^ may decrease apoptosis partially through this pathway.

In our study, in the I/R group, the expression of Beclin-1 was significantly increased and the number of neurons apoptosis was the highest. This phenomenon largely resulted from the proapoptotic function of Beclin-1, which was consistent with previously reported studies indicating the involvement of Beclin-1 in mitochondria-mediated apoptosis [8]. Recently, growing interest has focused on the role of Bcl-2 in autophagy, which is thought to be an important factor for crosstalk between autophagy and apoptosis [10, 32, 34]. Our results indicate the transition from autophagy to apoptosis might be via deregulation of Bcl-2 protein and upregulation of Bax protein. Therefore, we suggest that the neuroprotective mechanism of NAD^+^ is inhibiting the conversion of autophagy to apoptosis by increasing the phosphorylation of AKT/mTOR and decreasing the expression of Beclin-1. It is essential to comprehensively understand the complex function of autophagy and its relationship with apoptosis in SCIR.

Although we present important findings in this study, several limitations need to be addressed. First, although the SCIR model used in this study has been widely used due to the segmental blood supply of the spine, the model has its own shortcomings, such as lower limbs ischemia. Different modified models should be considered in future studies. Second, the period of postoperative observations was 24 h in our study. This period was not sufficient to determine whether NAD^+^ had long-lasting neuroprotective effects. In addition, NAD^+^ was administrated only at two predetermined doses (10 and 75 mg/kg) in this study, although our previous work has demonstrated NAD^+^ with the concentration higher than 50 mg/kg exerted a protective role in SCIR injury. Moreover, although no obvious side effects of 75 mg/kg NAD^+^ were identified until the predetermined observation time point, the lack of adverse response could be due to the limited observation time in our study; further studies with different dosages of NAD^+^ as well as longer observation time are needed to strengthen and extend our present work.

## 5. Conclusion

In summary, this study suggests excessive and sustained activation of autophagy in SCIR. Administration of NAD^+^ can decrease I/R induced injury and inhibit neuronal cell apoptosis. Further studies are needed to explore the implications of these findings.

## Abbreviations

I/R: Ischemia reperfusion
SCIR: Spinal cord ischemia reperfusion injury
TUNEL: Terminal deoxynucleotidyl transferase-mediated dUTP-biotin nick end labeling assay
LC3: Microtubule-associated protein 1 light chain 3
NAD^+^: Nicotinamide adenine dinucleotide
BBB score: Basso-Beattie-Bresnahan score
IOD: Integrated option density.
